# Young adults and multisensory time perception: Visual and auditory pathways in comparison

**DOI:** 10.3758/s13414-023-02773-7

**Published:** 2023-09-06

**Authors:** Giovanni Cantarella, Giovanna Mioni, Patrizia Silvia Bisiacchi

**Affiliations:** 1 Department of Psychology, University of Bologna, Viale Berti Pichat, 5, 40127 Bologna, Italy; 2 Department of General Psychology, University of Padova, Via Venezia, 8, 35131 Padova, Italy; 3 Padova Neuroscience Center, Padova, Italy

**Keywords:** Multisensory, Temporal bisection task, Healthy young adults, Timing, Perception

## Abstract

The brain continuously encodes information about time, but how sensorial channels interact to achieve a stable representation of such ubiquitous information still needs to be determined. According to recent research, children show a potential interference in multisensory conditions, leading to a trade-off between two senses (sight and audition) when considering time-perception tasks. This study aimed to examine how healthy young adults behave when performing a time-perception task. In Experiment 1, we tested the effects of temporary sensory deprivation on both visual and auditory senses in a group of young adults. In Experiment 2, we compared the temporal performances of young adults in the auditory modality with those of two samples of children (sighted and sighted but blindfolded) selected from a previous study. Statistically significant results emerged when comparing the two pathways: young adults overestimated and showed a higher sensitivity to time in the auditory modality compared to the visual modality. Restricting visual and auditory input did not affect their time sensitivity. Moreover, children were more accurate at estimating time than young adults after a transient visual deprivation. This implies that as we mature, sensory deprivation does not constitute a benefit to time perception, and supports the hypothesis of a calibration process between senses with age. However, more research is needed to determine how this calibration process affects the developmental trajectories of time perception.

## Introduction

Time perception can be considered one of the most crucial and pervasive aspects of human function (Grondin, 2010). As one of the first competencies to have evolved in biological systems, time perception has influenced the consequent development of almost all cognitive modalities (Gerstner, 2012; Paranjpe & Sharma, 2005).

A critical issue in the time perception field is the disentanglement of the plurality or singularity of temporal mechanisms (Grondin et al., 2008; Ivry & Schlerf, 2008). The conception of a single clock (Gibbon & Church, 1984; Grondin, 2001, 2010) appointed to this role dominated the field for a long period. Indeed, the Scalar Expectancy Theory (SET; Gibbon & Church, 1984) posited three basic levels of temporal representation: a pacemaker-accumulator internal clock, a level involving two sorts of memory (one essentially storing accumulator contents, and the other a ‘reference’ memory storing standards and other times important for the task at hand), and a decision process, which varies from one timing task to another. More recent intrinsic models have challenged this conception, implying that timing arises as part of modality-specific processing (Buonomano, 2000; Burr et al., 2007). In other words, do we have sensory-specific representations of time, or is there a centralised amodal mechanism (Bueti, 2011)?

### Sensory modalities for time perception

Time is not a physical stimulus, and, therefore, there is not a specific sensory receptor appointed to it (Grondin, 2010; Wearden, 2016). Nevertheless, a wide range of studies (Droit-Volet et al., 2004; Grondin, 1993; Grondin & Rammsayer, 2003; Grondin, 2005; Penney et al., 2000; Pütz et al., 2012; Walker & Scott, 1981; Wearden et al., 1998; Zélanti & Droit-Volet, 2012) have focused on the ways sensory modalities affect time-estimation performances.

Differences in estimated ranges and discrimination levels evidenced in distinct modalities represent a challenge to the hypothesis of a single clock responsible for time perception. In particular, the literature has indicated that the ability to process time depends on sensory inputs (Grondin & Rammsayer, 2003). In this perspective, several studies (Droit-Volet et al., 2004; Grondin & Rammsayer, 2003; Grondin, 2005; Pütz et al., 2012; Zélanti & Droit-Volet, 2012) have demonstrated that temporal intervals are perceived as lasting longer when stimuli are auditory, rather than visual, and that sensitivity to time is better (lower Weber Ratio (WR)) in the auditory modality.

When it comes to hearing, our ability to distinguish between sounds happening in quick succession is remarkably good. This is especially important for successive events (e.g., in speech or music) that need to be processed rapidly. Participants show better performances at discriminating intervals marked by auditory signals rather than by visual signals, and this finding can be applied to filled and empty intervals (Grondin, 1993). Intervals marked by auditory signals are perceived as longer than time intervals marked by visual signals (Grondin & Rammsayer, 2003; Penney et al., 2000; Walker & Scott, 1981; Wearden et al., 1998).

### Multi-modality and time perception

Behavioural evidence of the difficulty in achieving cross-modal transfer of temporal learning (Grondin et al., 2008) suggested the hypothesis of multi-modality for time perception. When considering the relative duration of intermodal intervals, an overestimation of intervals marked by an audiovisual sequence rather than intervals marked by a visual-auditory sequence (Grondin & Rousseau, 1991; Grondin et al., 1996) was highlighted. Indeed, presenting repeated standard intervals would enhance discrimination performance in the auditory modality (Drake & Botte, 1993); this does not necessarily occur in the visual modality if intervals are very short (e.g., in the range of 300 ms instead of 900 ms; Grondin, 2001). Penney et al. (2000) tested the effects of signal modality on duration classification by using a time-bisection task and selecting standard intervals in a timescale of seconds. During a test session, if auditory and visual signals share the same anchor durations, the visual signals are perceived as shorter than the auditory signals of the same duration.

Research has indicated that both transient and long-term sensory deprivations can impact time perception. This impairment may actually improve time estimation by reducing potential multisensory interference. Research conducted by Occelli et al. (2008), Stevens and Weaver (2005), Gori et al. (2014), Campus et al. (2019), and Opoku-Baah and Wallace (2020) support this finding. Experimental studies (Occelli et al., 2008; Stevens & Weaver, 2005) have investigated temporal aspects in blind adults with tactile, audio-tactile, and auditory stimuli, suggesting that the temporal performance of blind adults was more accurate than that of sighted adults when the auditory and tactile stimuli were presented from different positions rather than from the same position. Gori et al. (2014) employed an auditory time-bisection task but reported no significant differences between congenitally blind adults and the controls. Their results have been replicated more recently (Campus et al., 2019). Individuals with no sight would not receive more auditory stimuli than sighted individuals would; however, to interact more effectively with the environment, they should rely more on auditory inputs (Voss et al., 2008).

Opoku-Baah and Wallace (2020) induced a transient monocular deprivation during an audiovisual simultaneity judgement task, and this manipulation produced a narrowing of the temporal binding window, demonstrating the possibility to impact on audiovisual temporal perception. Nonetheless, the literature on the effects of transient sensory deprivation on time perception is still not exhaustive.

### The developmental profile of time perception

Research has highlighted a developmental trend for time perception: the mechanisms involved in temporal processing are present at an early stage, but their functioning improves with experience and maturation (Droit-Volet & Wearden, 2001; Droit-Volet et al., 2007; McCormack et al., 1999; Zelanti & Droit-Volet, 2011). The effect of signal modality on time perception with age was investigated by Droit-Volet et al. (2007) in 5- and 8-year-old children as well as young adults, using a time-bisection task in the time range of seconds. The modality effect was confirmed (higher overestimation in the auditory modality) in all samples; however, the magnitude of this difference was larger in the children than in the adults, suggesting a limitation of attentional abilities for both modalities. Moreover, they found an increasing time sensitivity with age that was explained as greater variability in the memory process (Droit-Volet & Wearden, 2001) underlying the representation of standard durations in the time range of seconds. Wearden and Jones (2013) confirmed previous findings that there is a decrease in variability in time estimation as individuals progress from childhood to adulthood. This supports the idea that attention, memory, and intellectual efficiency increased during this period. However, it's unclear if this also applies to the effects of transient sensory deprivation on time perception in the timescale of milliseconds.

### A trade-off between modalities in children

Battistin et al. (2019) investigated the influence of total or partial absence of sight on the time-estimation abilities of blind and visually impaired children. The study involved 63 children who were split into four different groups: blind, visually impaired, blindfolded, and sighted. All participants underwent an auditory temporal bisection task. Sighted children showed lower temporal abilities compared to the other groups. Moreover, interesting findings emerged from the blindfolded group: as well as the clinical groups, they showed higher accuracy in temporal judgements but no differences in temporal sensitivity compared to sighted children. The authors claimed that, in audiovisual conditions, the simultaneous presence of sight and audition led to a trade-off between the two senses, which was not present in the clinical groups or the blindfolded children because of their (congenital or transient) sensory deprivation. These results demonstrate that a congenital and transient condition of visual deprivation can effectively enhance auditory time-estimation abilities in children.

Furthermore, they seem to support using a single modality for better accuracy, independently of brain plasticity and reorganisation in blind people (Klinge et al., 2010; Weeks et al., 2000). However, considering a lower variability in temporal performances for young adults (Wearden and Jones, 2013), whether a transient visual deprivation could also enhance time-estimation abilities in a student-age cohort of participants is not yet known. Additionally, the effects of auditory deprivation on the time-estimation abilities of young adults are still unexplored.

Taken together, the results of the studies investigating the role of multi-modality in time perception and the developmental profile of this ability are inconclusive, suggesting that commonalities (or differences) of timing mechanisms across different sensory modalities are still a matter of debate.

### The present research

Based on previous research (Battistin et al., 2019, Campus et al., 2019; Gori et al., 2014; Penney et al., 2000), the present study is one of the first to investigate separately the impact of transient sensory deprivation on young adults' time-estimation abilities, also comparing these abilities to those of children in the same conditions (with and without transient sensory deprivation).

The novelty is in the attempt to address the following questions: To what extent could a condition of (both auditory and visual) transient deprivation affect time-estimation performances of young, healthy adults in the timescale of milliseconds? Is there a developmental profile according to which the impact of transient sensory deprivation on time estimation changes with age?

## Experiment 1

The same subjects learned standard durations separately in the visual and auditory modalities in two experimental sessions. Within each session, a condition of transient auditory deprivation (with visual stimuli) or visual deprivation (with auditory stimuli) was implemented. We investigated whether the induction of a transient visual or auditory deprivation could affect their temporal judgements.

As occurred in blindfolded children (when compared to sighted peers; Battistin et al., 2019), we expected that transient sensory deprivation would enhance time-estimation performances in the auditory modality. Indeed, amplifying the processing of temporal stimuli in the preserved sensory modality would facilitate the duration perception. Participants should overestimate the perceived duration in the auditory modality, as the internal clock runs at a faster rate (Grondin & Rammsayer, 2003; Penney et al., 2000; Walker & Scott, 1981; Wearden et al., 1998). We would also expect a better sensitivity for auditory modality in time-discrimination performance, in agreement with most research on this topic (Grondin, 1993, 2005; Grondin et al., 1998; Tallal et al., 1993). Given the absence of clear results in the literature regarding this effect, we did not have strong a priori expectations about the effect of a transient auditory deprivation on visual time-estimation abilities.

## Methods

### Participants

Young, healthy adults were recruited from the local community and were tested individually. The sample was originally composed of 56 experimental subjects; 55 subjects were considered for statistical analyses (16 males and 39 females). They were healthy adults of Italian nationality, aged between 19 and 27 years (mean age = 22.11 years; SD = 2.20). In most cases (96%) participants were right-handed (53), which was quantified (mean = 47.44) by using the Handedness Edinburgh Inventory (Oldfield, 1971).

We had to exclude one participant's data from our analysis because they did not follow the task instructions properly. This participant's data were considered an outlier. The likelihood of having experienced head trauma or epilepsy was assessed as exclusion criteria to perform statistical analyses on the collected data.

The study took place at the Department of General Psychology in Padua, Italy. All participants willingly gave their consent to take part in the research and were informed that their participation was voluntary. They were also made aware that they could stop the testing at any point. Monetary compensation was given after each session.

### Procedure

We created a within-subjects design, whereby each subject was administered the same task. We adapted the experimental design for both visual and auditory modalities, using either visual or auditory signals to train them. This helped us determine how much each sensory channel contributed to their performance. The study involved two experimental sessions conducted on separate days: one for visual and one for auditory tasks. Each session had two conditions – one with sensory deprivation (a) and one without (b) – to see if using a single sense could improve time estimation. To ensure that practice effects did not influence the results, the conditions were counterbalanced in various orders of administration. Each participant could randomly begin with one of the two sensory modalities in (a) or (b) conditions. During each session, participants were able to complete the bisection task with and without sensory deprivation in any order, resulting in a total of eight possible combinations.

Participants sat in front of an Intel-based, 64-bit Windows PC (85-Hz refresh rate, 60-cm distance between participants and the monitor) running Windows 7, connected to a high-resolution monitor in a quiet room (a silent cabin). For the auditory mode, we provided headphones specifically for audio. To induce sensory deprivation, we introduced noise-cancelling headphones and earplugs. Additionally, each subject wore a mask to block out visual stimuli. We consistently tested the effectiveness of these measures in inducing sensory deprivation rather than just muffling sound and blocking vision.

Along with the presentation of experimental procedures, E-Prime 2.0 software was used to set up the sequence of visual and auditory stimuli. The local ethics committee approved this procedure (Protocol Code 2116).

#### Time-bisection task

The time-bisection task (Kopec & Brody, 2010) was composed of four experimental blocks. The task was divided into a learning phase and a testing phase.

In the learning phase, 10 short (S = 300 ms) and 10 long (L = 900 ms) standard durations were administered. There was only a single learning period at the beginning of each experimental session. As declared in previous studies (Kopec & Brody, 2010; Penney & Cheng, 2018), short standards were presented first in each learning phase. The auditory stimulus was a pink noise, which was independently generated for each trial; the visual stimulus was a black circle (size: 4.5 cm) that appeared on a white screen. Both standard durations were presented ten times in the learning phase so participants could memorise them. A test phase was conducted, which involved comparing two standards for seven different durations (300, 400, 500, 600, 700, 800, and 900 ms). Subjects were required to judge the relative durations of new intervals and to determine whether they were closer in duration to the ‘short standard’ or the ‘long standard’.

The task was split into four blocks: in each block, each duration (300, 400, 500, 600, 700, 800, and 900 ms) was presented seven times for 49 trials. Responses were recorded by pressing (with the right or left index finger) one of two keys on the PC keyboard (‘A’ or ‘L’), according to the time judgement. After each response, there was a 1,000-ms inter-trial interval. Subjects were not provided with feedback about the accuracy of their responses.

### Data analyses

Temporal abilities were first analysed in terms of the proportion of long responses (raw data), which consisted of the relative proportion times each subject pressed ‘long’ for each new comparison interval considered. Consequently, an overall seven-point psychometric function was traced, plotting the seven comparison intervals on the x-axis and the probability of responding ‘long’ (p-long) on the y-axis for each experimental condition.

The bisection point (BP) was calculated for each participant. BP is defined as the stimulus duration for which the participants responded ‘short’ or ‘long’ with equal frequency. The BP is associated with the target duration corresponding to a predicted rate of long responses of 50%, and it is used as a measure of perceived duration: the smaller the BP value, the longer the perceived duration.

Temporal abilities were also analysed in terms of Constant Error (CE): it is defined as the duration of the mid-point between the two standards (300 and 900 ms) minus the BP (Grondin et al., 2015). CE is a measure of accuracy positively related to perceived duration. Positive or negative CE values are an index of over- or underestimation of temporal durations compared to the mid-point.

In addition, the WR parameter was implemented. It is defined as the degree of discriminability the subject uses to parse the standard durations into the ‘short’ and ‘long’ categories. This variable measures the participant's sensitivity to time: a subject with a high degree of discriminability would produce a psychometric curve that appears very step-like, resulting in a low WR, while a poorer discriminability would result in a more gradual psychometric function and a higher WR (Kopec & Brody, 2010). WRs were calculated as the ratio of the just noticeable difference (JND; half of the difference between the intervals giving 25% and 75% of the psychometric function) to the correspondent standard interval (ranging from 300 to 900 ms). For each participant, discrimination sensitivities for visual and auditory modalities were estimated separately (Table 1 summarizes the descriptive statistics for BP, CE and WR values).

**Table 1:** Descriptive statistics for bisection point (BP), constant error (CE) and Weber Ratio (WR) as a function of conditions. Values are given as mean (SD)

	Visual	Visual with deprivation	Auditory	Auditory with deprivation
*BP*	599 (89)	581 (91)	520 (62)	520 (65)
*CE*	0.95 (89)	19 (91)	84 (60)	85 (60)
*WE*	0.28 (0.09)	0.30 (0.13)	0.18 (0.07)	0.19 (0.07)

The order of administration among modalities (auditory–visual or visual–auditory) was considered as a between-subjects factor to investigate the role of potential practice effects among sessions.

Since the data distributions for both BP and CE values did not violate the assumption of normality (*p*s > .25 in most cases), we conducted parametric tests (ANOVAs) on these indices. A repeated-measures ANOVA was performed, considering BP as a dependent variable, with order of administration as a between-subjects variable and modalities (auditory or visual) and deprivation (presence or absence) as within-subjects factors. We conducted a repeated-measures ANOVA on CE. However, the normality assumption was violated for the distribution of WR values (Shapiro-Wilk test; all *p*s < .005). Therefore, we performed a non-parametric analysis (Friedman test) on these measures separately for visual and auditory modalities as well as conditions of sensory deprivation (presence or absence). Additionally, we used the Mann-Whitney test to perform another non-parametric analysis on WRs with the order of administration as a between-subjects factor. 

## Results

### Descriptive results for the proportion of ‘long’ responses

**Fig. 1:**
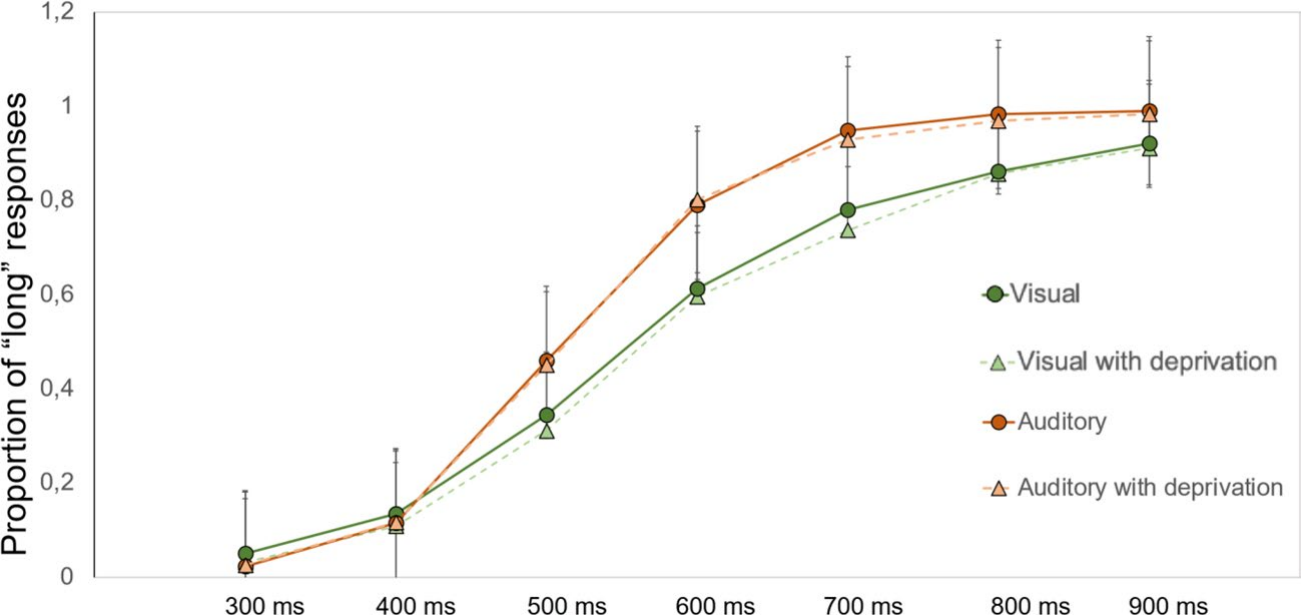
Proportion of ‘long’ responses as a function of Conditions and Intervals. Error bars indicate ± standard deviations from the mean

Considering the seven-point psychometric function (see Fig. 1), it appears that subjects tend to overestimate the auditory modality compared to the visual one. Within each modality, no overall differences are detectable when manipulating the presence of sensory deprivation.

### Results for bisection point (BP)

**Fig. 2:**
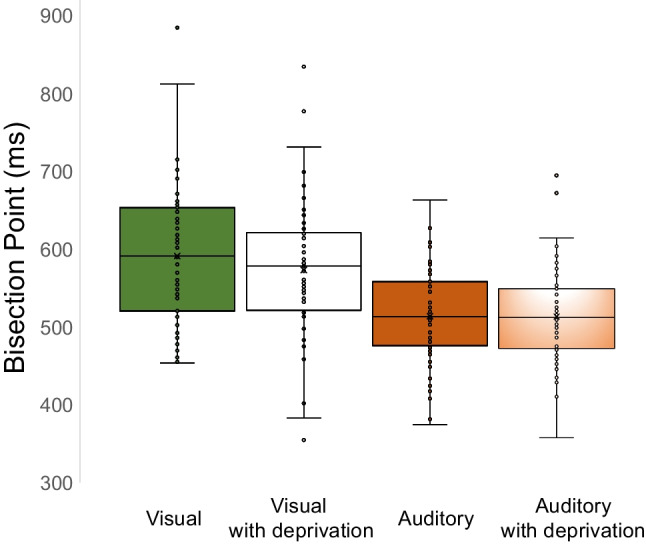
Boxplots illustrating the distribution of bisection point values across conditions. The box represents the interquartile range, with the median indicated by the horizontal line inside the box. Outliers are represented by individual data points

A repeated-measures ANOVA conducted on BP showed a main effect of modalities [*F* (1.53) = 66.25, *p* < .001, η^2^_p_ = .556] (see Fig. 2).

The variable deprivation [*F* (1.53) = .935, *p* = .338, η^2^_p_ = .017] and the interaction Deprivation × Modalities [*F* (1.53) = 1.71, *p* = .197, η^2^_p_ = .031] did not reach statistical significance. When considering a different order of administration as a between-subjects factor, the absence of a statistically significant effect was shown [*F* (1.53) = .776, *p* = .382, η^2^_p_ =.014] among performances.

### Results for constant error (CE)

**Fig. 3:**
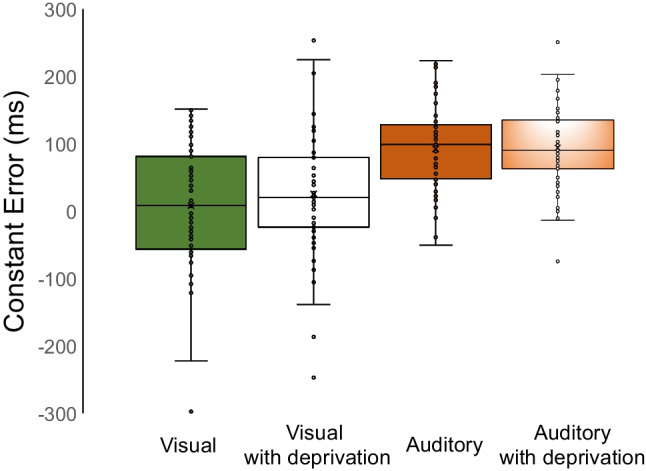
Boxplots illustrating the distribution of constant error values across conditions. The box represents the interquartile range, with the median indicated by the horizontal line inside the box. Outliers are represented by individual data points

A repeated-measures ANOVA conducted on CE yielded a main effect of modalities [F (1.53) = 69.893; *p* < .001; η^2^_p_ = .569] (see Fig. 3).

The variable deprivation [*F* (1.53) = 1.197, *p* = .279, η^2^_p_ = .022] and the interaction Deprivation × Modalities [*F* (1.53) = 1.357, *p* = .249, η^2^_p_ = .025] failed to reach statistical significance. The absence of a statistically significant effect was also shown when considering a different order of administration [*F* (1.53) = 1.008, *p* = .320, η^2^_p_ =.019] among performances.

### Results for Weber Ratio (WR)

**Fig. 4:**
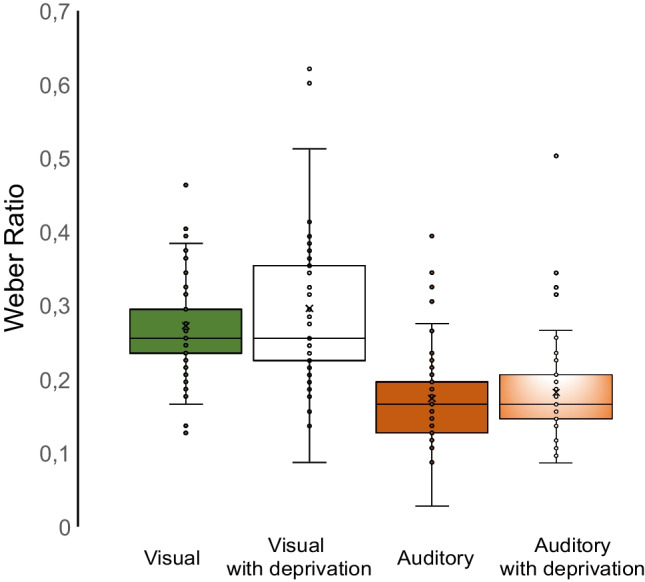
Boxplots illustrating the distribution of Weber Ratio values across conditions. The box represents the interquartile range, with the median indicated by the horizontal line inside the box. Outliers are represented by individual data points

The Friedman test conducted on WR as a dependent variable yielded a main effect of modalities [chi-squared= 57.899, W = .704; *p* < .001] (*see* Fig. 4).

The variable deprivation [chi-squared= .187; W= .026; *p* = .665] did not reach statistical significance. Results of the non-parametric analysis (Mann-Whitney test) on WRs showed the absence of statistically significant differences (all *p*s ≥ .084) between different orders of administration.

## Interim discussion

In our study of young adults, we found that they were able to accurately differentiate time durations. This was shown by an increase in the proportion of longer responses as the actual length of the duration being judged increased, which suggests that they followed the task instructions properly in both auditory and visual modes. We found a systematic tendency to overestimate time intervals (higher BPs) in the auditory modality, which led to lower accuracy in time estimation (higher CEs) compared to the visual modality. Our findings also pointed to a better time sensitivity (lower WRs) in auditory time bisection when compared to the visual modality. However, we did not observe any differences in time estimation between visual and auditory modalities in the presence or absence of transient sensory deprivation. These results were unexpected and contrary to our hypothesis, which suggested that sensory deprivation would benefit time estimation in young adults as it does in children (Battistin et al., 2019). These results suggest that there may be a calibration process between these two senses with age, which could eliminate the trade-off between audition and sight, optimizing time estimation in multi-sensory conditions.

## Experiment 2

Since we were interested in testing how accuracy in temporal abilities and time sensitivity change with increasing age, two samples of children (sighted and sighted but blindfolded) were selected from a previous database (Battistin et al., 2019) and their temporal abilities (i.e., CE) were compared to that of our sample of young adults in the two corresponding conditions (auditory and auditory with deprivation). This allowed us to directly test for the occurrence of a calibration process between sight and audition with increasing age, from a maturational point of view.

As occurred in previous research investigating the time-range of seconds (Droit-Volet et al., 2007), we expected a maximization of the modality effect, with a higher overestimation of auditory stimuli in the sample of children versus young adults, and an improvement of time sensitivity (i.e., lower WR) with age. We also expected a differential impact of a transient visual deprivation, depending on age: in view of an optimal multisensory integration, young adults (as opposed to children; see Battistin et al., 2019) should not benefit from a transient deprivation to estimate time, and this deprivation should not affect their temporal abilities.

## Methods

### Participants

Two groups of children were selected from a previous database (Battistin et al., 2019). Twenty children were sighted (males = 7; mean age = 9.05 years; SD = 1.19) and performed the auditory temporal bisection task without any sensory deprivation; 16 were sighted as well but performed the task completely blindfolded (males = 10; mean age = 9.31 years; SD = 1.14), so with the induction of a transient visual deprivation.

The experimental conditions occurring for these children were identical (see Battistin et al., 2019) to those of young adults in Experiment 1: they performed a time-bisection task in the auditory modality, with standard durations in the time-range of milliseconds (from 300 ms to 900 ms). It was composed of a learning phase and a test phase (see above, Experiment 1).

### Data analyses

This comparison was performed only for time-estimation performances in the auditory modality (with visual deprivation or not) because previous data of (sighted and sighted but blindfolded) children were available only for these conditions. We considered their performances at the auditory time-bisection task, classified in terms of BP (perceived duration), CE (accuracy in time estimation) and WR (time sensitivity).

#### Auditory

An ANOVA was conducted on BP values calculated in the auditory modality, with group (young adults vs. sighted children) as a between-subject factor. The same analysis was run on CE values. As the distributions of WRs for both groups violated the assumption of normality, a non-parametric test (Mann-Whitney) was performed to compare children versus adults on these measures.

#### Auditory with deprivation

An ANOVA was conducted on BP values calculated in the auditory modality with visual deprivation, by considering group (young adults vs. sighted blindfolded children) as a between-subject factor. The same statistical approach was employed for CE values. A non-parametric Mann-Whitney test was performed instead to compare WR measures between groups.

## Results

### Results for BP

**Fig. 5:**
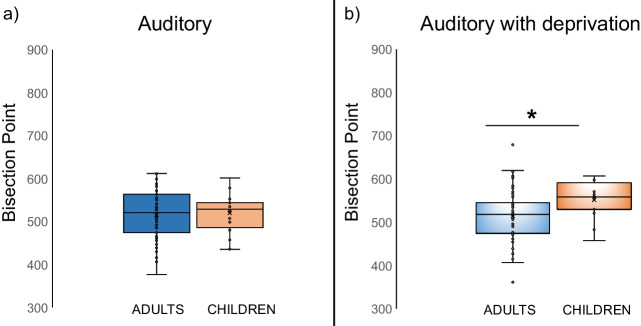
Boxplots illustrating the distribution of bisection point values across groups (adults vs. children). The box represents the interquartile range, with the median indicated by the horizontal line inside the box. Outliers are represented by individual data points. (**a**) *condition*: auditory; (**b**) *condition*: auditory with deprivation

Regarding the ANOVA on BP values (*condition*: auditory) with *group* as between-subject factor, the factor *group* did not reach statistical significance [F (1.73) = .095; p= .758]*;* see Fig. 5a.

Instead, the same ANOVA on BP values (condition: auditory with deprivation) yielded a main effect of *group* [F (1.69) = 5.431; p = .023; η^2^_p_ = .073], with blindfolded children showing higher BP values (less overestimation) compared to blindfolded adults*;* see Fig. 5b*.*

### Results for CE

**Fig. 6:**
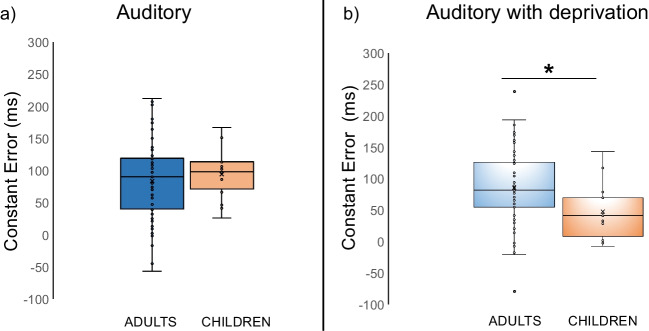
Boxplots illustrating the distribution of constant error values across groups (adults vs. children). The box represents the interquartile range, with the median indicated by the horizontal line inside the box. Outliers are represented by individual data points. (**a**) *condition*: auditory; (**b**) *condition*: auditory with deprivation

When considering the ANOVA conducted on CE (*condition*: auditory) with *group* as between-subject factor, the factor *group* failed to reach statistical significance [*F* (1.69) = 0.420, *p* = .519, η^2^_p_ = .006]; see Fig. 6a*.*

However, the same ANOVA conducted on CE (*condition*: auditory with deprivation) with *group* as a between-subject factor showed a main effect of *group* [*F* (1.69) = 5.431, *p* = .023, η^2^_p_ = .073]. It revealed a lower CE (and better temporal performances) in a sample of blindfolded children when compared to our sample of blindfolded adults; see Fig. 6b.

### Results for WR

**Fig. 7:**
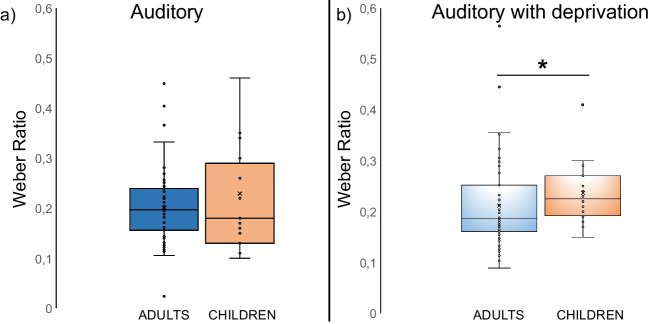
Boxplots illustrating the distribution of Weber Ratio values across groups (adults vs. children). The box represents the interquartile range, with the median indicated by the horizontal line inside the box. Outliers are represented by individual data points. (**a**) *condition*: auditory; (**b**) *condition*: auditory with deprivation

The Mann-Whitney test conducted on WR (*condition*: auditory) resulted in the absence of statistically significant differences (H = 555.00; p = .526) among groups; see Fig. 7a.

However, the same Mann-Whitney test performed on WR (*condition:* auditory with deprivation) showed the presence of statistically significant differences among the two groups (children vs. adults) (H = 309.50; p = .037). WR values were higher (i.e., a lower time sensitivity) in the sample of blindfolded children compared to the sample of blindfolded adults; *see* Fig. 7b.

## Interim discussion

This second set of analyses comparing the temporal abilities of young adults versus children revealed different behavioural patterns depending on the condition (auditory vs. auditory with deprivation). No differences were found among groups when judging auditory intervals under conditions without sensory deprivation. Instead, transient visual deprivation differentially affected temporal abilities in both groups: blindfolded children had higher accuracy values in temporal judgements (lower CE) while young adults did not benefit from this deprivation. Moreover, visually deprived adults had a higher time sensitivity (lower WR) compared to blindfolded children, confirming an improvement in time sensitivity with age. These results suggest the possibility for a calibration process among the visual and auditory senses with age and its effects, bound to a condition of transient sensory deprivation, on time-estimation abilities.

## General discussion

The purpose of this research was to investigate whether temporary sensory deprivation affects time perception in young adults. To achieve this, we conducted two experiments. In the first experiment, the same group of participants completed visual and auditory time tasks separately in two different experimental settings. In each session, they were either visually deprived of auditory stimuli or auditorily deprived of visual stimuli. In the second experiment, we compared the temporal abilities of young adults with those of two groups of children from a previous study (Battistin et al., 2019) under the same conditions. We hypothesized that transient sensory deprivation would improve time estimation among young adults, as observed in children. However, our results did not support this hypothesis.

Our main finding was that transient sensory deprivation does not affect the ability of young adults to estimate time, contrary to the results observed in children. This suggests that as people age, a calibration process between vision and hearing may occur, which affects time estimation under sensory-deprivation conditions. This process is further explained in the following sections.

### The achievement of an optimal multisensory integration with age

Our sample of young adults did not show differences in accuracy (CE) after a transient sensory (visual or auditory) deprivation (Experiment 1). Moreover, in conditions of visual deprivation, they revealed a higher tendency to overestimate (lower BP) at the auditory bisection task compared to a sample of blindfolded children: the use of a single modality did not reduce the bias towards responding long, as occurred for children (Experiment 2).

In Experiment 2, age effects occurred in the condition of visual deprivation only, but not under conditions without sensory deprivation, where young adults and children showed instead comparable temporal abilities (for BP – perceived duration, CE – temporal accuracy and WR – time-sensitivity). However, it is worth noting that our sample of children (mean age = 9.05 years), despite being the maturational process of calibration among senses (that usually appears at 10 years of age; see Droit-Volet, 2013; Droit-Volet and Coull, 2016; McCormack et al., 1999*;* Zelanti & Droit-Volet, 2011) still in progress, could have reached sufficient calibration levels among senses and stability of memory representations for durations to achieve the precision of young adults’ temporal judgements under the same conditions (without transient sensory deprivation). Alternatively, the higher variability of the sighted children in temporal judgements (e.g., see Fig. 7, showing the data distribution; see also Droit-Volet & Wearden, 2001) compared to the blindfolded children could have abolished the occurrence of their behavioural peculiarities as well as potential emerging differences with young adults in these conditions. As the performance distribution of sighted children was much more variable than those of the young adults, this could have increased the within-group variance and consequently decreased the between-groups variance detected by the statistical test.

On the other hand, conditions of visual deprivation may have maximized the contribution of the remaining sensory modality in time judgements (in this case, at greater expense to the auditory modality), partially explaining the age effect. For example, the exclusive use of the auditory modality to encode temporal stimuli in the transient visual deprivation condition may have improved the performance of children by reducing the bias towards temporal overestimation (higher BP) that was present instead in the sample of young adults in the same condition. Consistently, this effect resulted in more accurate temporal judgments (lower CE values) and enhanced in blindfolded children compared to blindfolded adults.

Such discrepancies in timing performances can be interpreted from a maturational point of view. Indeed, a calibration process between sight and audition throughout the participants’ lifetimes may be necessary to achieve an optimal multisensory integration between sight and audition in time estimation. This achievement would lead healthy adults to combine information from different sensory modalities for time estimation simultaneously. Hence, transient sensory deprivation would hinder this likelihood of integrating audiovisual signals to estimate time. On the other hand, blindfolded children could benefit more from visual deprivation (lower CE, i.e., less overestimation and higher accuracy) because, at their age, the maturational process of calibration between auditory and visual signals for time processing is not yet complete.

At which stage of human development would this multisensory integration process occur? A recent study investigated developmental trajectories of multisensory integration (Adams, 2016) by using an audiovisual counting task, in which, for each trial, observers were presented with several beeps and/or flashes. In separate blocks, the observers reported either the number of flashes or the number of beeps. Their results demonstrated that optimal audiovisual integration emerges in 10 years. Before this age, children do not integrate audiovisual information, but they switch between using only auditory or only visual information on each trial (Adams, 2016). Moreover, the ability to integrate audiovisual modalities develops at a similar age to integration across and within other modalities. One previous audiovisual study (Gori et al., 2012) with children aged 5–14 years and adults failed to find optimal integration at any age. This study employed a time-bisection task, in which observers estimated which of two empty intervals was longer. Subsequent work (Hartcher-O'Brien et al., 2014) has shown that, for this type of task with empty intervals, observers integrate auditory and visual information to estimate the time points at the ends of the interval optimally, rather than integrating duration per se. With filled intervals, optimal integration of duration estimates would likely be found with children aged around 10 years, as it is in adults. Why does this ability fail to appear until approximately 10 years of age? One proposed explanation is that the lack of integration is beneficial during early childhood and facilitates recalibration (Gori et al., 2008; Nardini et al., 2010). During this period of growth and sensory development, constant sensory recalibration is required to maintain accurate (unbiased) perceptual estimates (Adams, 2016).

As well as a stronger tendency to overestimate, our sample of blindfolded adults showed a better time sensitivity (lower WR) in the auditory version of the bisection task concerning the sample of blindfolded children. This result, while not so statistically strong and therefore to be interpreted with caution, points to the evidence that such a calibration process between senses, with age, would also determine an improvement in the degree of discriminability between durations (i.e., time sensitivity) in conditions of transient sensory deprivation.

Why would children need to improve their time sensitivity with age? Misjudgements of time have been found in several studies with young children (Droit-Volet and Zélanti, 2013, Droit-Volet and Coull, 2016), and they have been linked to the children’s limited cognitive abilities. According to the ‘internal clock’ models (Gibbon & Church, 1984), time misjudgements have been classified as deficits to cognitive modules added to the ‘clock stage’ as part of a wider temporal information processing. For instance, time distortions have been explained by the time units emitted by the clock not being entered into a person’s memory due to a lack of attention toward time (Zakay & Block, 1996). The modelling indicated increasing timing sensitivity as the children grew older, accompanied by a reduction in responses not controlled by stimulus duration, which could be linked to increasing attentional capacity (Zelanti & Droit-Volet, 2011). Such results have also been explained by memory loss when the retention interval increases (Droit-Volet et al., 2007) and by a ‘noisier’ memory representation of the standard durations in reference memory due to a less efficient learning process (Droit-Volet & Wearden, 2001). The magnitude of the signal modality’s impact on the subjective experience of duration decreases with development, with a greater amount of noise in time encoding for visual signals, rather than for auditory signals, in young children. A parallel increase in time sensitivity was also found, as the maturational process was not complete, at the age of 8 years (Droit-Volet et al., 2007). Accordingly, developmental trends highlighted memory/timing variability decreases, and the proportion of ‘random’ responses declined with increasing age, nearing zero in 8-year-old children. These results were similar to those obtained in an earlier paper by McCormack et al. (1999), who tested children of 5, 8 and 10 years of age on a bisection task. Their ‘noise’ parameter in the encoding stage declined systematically in value with age: Comparisons with data from adults suggest that student-age adults have the smallest variability values (steeper psychometric function, as occurred for our sample of young adults; see above, in the *Results* section). A greater amount of noise in the time encoding of young children could be due to their limited attention capacities, that is, their difficulty in maintaining attention during the continuous duration perception. There seems to be a ‘coherent’ decline in variability, which coincides with the idea that attention, memory and general intellectual efficiency tend to increase from early childhood to early adulthood (Wearden & Jones, 2013). Thus, a more stable representation of time and an increase of attentional resources, in visual and auditory modalities, lead healthy adults to having better time sensitivity following transient visual deprivation.

### Behavioral peculiarities of young healthy adults’ performance on time estimation across modalities

The present study confirmed an overestimation in the auditory modality when compared to the visual one, in agreement with most of the field’s literature (Droit-Volet et al., 2004; Grondin & Rammsayer, 2003; Grondin, 2005; Zélanti & Droit-Volet, 2012). This result can be contextualized within the Scalar Expectancy Theory’s framework (Gibbon & Church, 1984): The modality difference could be conceived because of clock-stage and memory-stage mechanisms. A possible explanation is that an internal clock runs at a faster rate for auditory signals than for visual ones. Therefore, the accumulated clock value for a given duration is larger when the signal is auditory than when it is visual. As a result, if the auditory and visual accumulations are compared, the auditory signal will seem longer. Alternatively, auditory signals may be more readily processed than visual ones are, meaning that the auditory–visual difference is due to a latency difference in the initiation of timing (Jaskowski et al., 1990).

In our sample, a better time sensitivity in the auditory modality, concerning the visual modality, was highlighted. Several studies support this idea, showing that the auditory system is the most accurate one to represent temporal information, but vision is crucial for spatial representation (Barakat et al., 2015; Bresciani & Ernst, 2007; Burr et al., 2009; Guttman et al., 2005). Recently, it was confirmed that the brain uses auditory representations to deal with complex temporal representations across multiple sensory modalities (Amadeo et al., 2020). Better performance on auditory duration discrimination is generally ascribed to an increased number of pulses accumulated during a given time interval in the case of auditory stimuli compared to visual stimuli. This increased number of pulses yields finer temporal resolution and, thus, better time sensitivity for auditory time intervals (Rammsayer et al., 2015).

The order of administration alone did not affect performances between modalities. Nevertheless, in auditory–visual order, the tendency to a worsening in visual performances (WRs) was shown. Auditory performances evidenced better stability among the orders of administration. This result could be contextualized to the extent to which auditory and visual stimuli contribute to the perception of durations in the range of milliseconds. The question is to understand to what degree selective attention modulates the influence of each modality. In this regard, research has shown auditory signals dominate in time perception when a stimulus is redundantly presented through auditory and visual modalities. Thus, time perception predominantly depends on auditory signals, regardless of the relative salience of the auditory and visual signals (Ortega et al., 2009). Recently, researchers have speculated that the brain uses auditory representations to deal with complex temporal representations across multiple sensory modalities (Amadeo et al., 2020). However, future researchers should investigate the cortical activations that are involved in temporal representation by employing additional unisensory or multisensory contexts.

### Limitations and future directions

Our results of a modality effect, when signals are presented in two separate sessions, seem to contradict a previous study (Penney et al., 2000). In this case, the modality effect occurred only when visual and auditory signals were presented in the same test session and shared the same anchor durations (a timescale of seconds). A model that posits across-modality memory with a temporal accumulation difference was hypothesized to account for the pattern of results. The memory representation may be thought of as an average to which the accumulations on each anchor trial for each modality contribute (Penney et al., 2000). Within this framework, the pacemaker-accumulator module of the clock process is differentially driven by different signal modalities, and the auditory modality dominates the reference memory process that mixes the modality standards. A contradiction in our results could be due to the time range selected (in our study, a timescale of milliseconds) and to the within-subjects experimental design (the same participant performing two separate test sessions). Indeed, the latter (Walker & Scott, 1981; Wearden et al., 1998) is a common feature of most studies finding a modality effect.

We did not reveal statistically significant differences among groups (adults vs. children) under conditions without sensory deprivation. This finding seems to be in contradiction to Droit-Volet et al. (2007), pointing instead to a maximization of modality effect and a better time sensitivity with age. Nonetheless, it’s worth saying that both visual and auditory stimuli, in our experimental paradigm, ranged from 300 to 900 ms. It’s likely that, in the time-range of milliseconds, the greater amount of noise in the time encoding of children and their limited attention capacities would not play a role in determining time-estimation performances. The internal clock with the accumulation of pulses by the pacemaker (Gibbon & Church, 1984) and the representation of sensory stimuli to be discriminated (Amadeo et al., 2020) could rather be involved. These factors, pertaining to the maturation of dedicated timing components per se, do not seem to be influenced by age (Droit-Volet et al., 2007).

Sensory integration has the potential to provide benefits for virtually all everyday activities: precision is improved by combining redundant information sources either within or across modalities. The importance of multisensory interaction for sensory calibration and development is supported by studies (Gori et al., 2010, 2012) in populations with sensory impairments, and it can be considered a source of undoubted clinical-operative implications. An example would be to consider the interconnections between temporal skills and motor coordination. Indeed, the localization of the sense of space, as a perceptual function, would depend on a ‘calibration’ process (cross-sensory facilitation) between visual and auditory modes (Gori et al., 2011). In addition, individuals who experience deprivation seem to employ time information encoded to infer spatial coordinates of the environment (Gori et al., 2018). The possibility to reinforce perceptual development has been demonstrated with blind (or visually impaired) children through the introduction of audio-motor feedback that acts as an aid to the coordination of their movements in the surrounding space (Cappagli et al., 2019).

## Conclusions

We demonstrated that transient sensory deprivation does not improve the time-sensitivity of healthy adults in visual and auditory modalities. We also found that transient visual deprivation decreases the accuracy of temporal abilities and increases the time sensitivity of blindfolded children, but not for young adults in the same condition. This result can be interpreted as evidence of the possibility that, in adulthood, audiovisual integration may not constitute an obstacle to time estimation. Indeed, an optimal multisensory integration between visual and auditory signals would maximize the processing of temporal intervals to be estimated. On the other hand, children could not benefit from this calibration process among the senses (Battistin et al., 2019), benefiting from transient sensory deprivation to correctly process time durations. The evidence that sensory deprivation did not constitute a benefit with age and the hypothesis of a calibration process between senses to optimize multisensory integration in time estimation highlights the necessity of investigating additional human peculiarities in this field.

## Data Availability

The datasets generated and/or analysed during the current study are available on the project’s Open Science Framework page (osf.io/7uszj).
